# Assessment of the predictive role of pretreatment Ki-67 and Ki-67 changes in breast cancer patients receiving neoadjuvant chemotherapy according to the molecular classification: a retrospective study of 1010 patients

**DOI:** 10.1007/s10549-018-4730-1

**Published:** 2018-02-26

**Authors:** Rui Chen, Yin Ye, Chengcheng Yang, Yang Peng, Beige Zong, Fanli Qu, Zhenrong Tang, Yihua Wang, Xinliang Su, Hongyuan Li, Guanglun Yang, Shengchun Liu

**Affiliations:** Department of Breast Surgery, The First Affiliated Hospital of Chongqing Medical University, No.1 Youyi Road, Yuzhong District, Chongqing, 400042 China

**Keywords:** Breast cancer, Tumor response, Molecular subtypes, Neoadjuvant chemotherapy, Ki67

## Abstract

**Purpose:**

To assess the predictive role of pretreatment ki67 and Ki67 changes in breast cancer (BC) patients treated with neoadjuvant chemotherapy (NAC) in various molecular subtypes.

**Methods:**

1010 BC patients who had undergone anthracycline and taxane-based NAC from January 2012 to July 2017 were retrospectively analyzed. Clinical and pathological parameters of the patients were retrieved and the predictive factors for NAC response were evaluated.

**Results:**

705 patients showed clinical response (cRes), and 131 patients acquired pathologic complete response (pCR). Patients with higher pretreatment Ki67 (≥ 14%), tumor size ≥ 4 cm, and positive clinical nodal had better clinical therapy response, while patients with negative ER and PR, higher pretreatment Ki67 (≥ 14%), and tumor size < 4 cm were more probable to attain pCR. The pretreatment Ki67 could be used as a predictor of NAC only in luminal subtypes, and 25.5% were identified as an ideal cut-off point to differentiate the cRes from non-cRes cases. Although a decrease in Ki67 had been found in almost all molecular subtypes after NAC, no statistically significant differences were found in the decrease of Ki67 were validated between the cRes and non-cRes group in HER2-rich and triple-negative subtypes (*P* = 0.488 and *P* = 0.111, respectively).

**Conclusions:**

The best cut-off for pretreatment Ki67 in predicting the connection with the tumor size lessening was 25.5% in luminal subtype. Aggressive adjuvant systemic treatments should be considered for patients with HER2-rich and triple-negative subtype who exhibit tumor shrinkage in NAC but still have high levels of Ki67.

**Electronic supplementary material:**

The online version of this article (10.1007/s10549-018-4730-1) contains supplementary material, which is available to authorized users.

## Introduction

Neoadjuvant chemotherapy (NAC) is a standard treatment for advanced breast cancer (BC) patients with the aim to decrease the extent of surgery [1]. Moreover, it is possible to evaluate the efficacy of NAC in a comparatively short time via therapeutic response, which lets tumor response to chemotherapeutic agents be monitored by this approach [2]. Based on the 2011 St Gallen consensus, there are four subtypes of BC: luminal, luminal–HER2, HER2-rich, and triple-negative on the basis of the immunohistochemistry results of ER, PR, and HER2 [3]. Many studies have revealed effective predictors of the response to NAC with different molecular subtypes [4–6], but some of these conclusions remain controversial.

ki67 level was related to tumor cell proliferation, which is the first immunohistochemical (IHC) marker that calls for a precise quantity [7]. A great number of studies have shown that Ki67 was regarded as the marker that provides prognosis for BC patients who have undergone NAC [8–10], and it has been observed that Ki67 is a factor that can predict the response to NAC [7, 11, 12]. However, despite the increasing evidence showing the predictive value according to molecular subtype, it is not clear whether Ki67 is identically helpful for predictive approaches in various subtypes, especially the changes of Ki67 during NAC.

In this clinical practice, we have evaluated whether pretreatment Ki67 levels are able to calculate the effectiveness of chemotherapy among molecular subtypes with the same chemotherapy regimen. Furthermore, we investigated whether the change of Ki67 between the needle biopsy and the residual tumor can be used as a predictor for NAC in different subtypes with a relatively large number cohort. In addition, the clinical and pathological response of NAC was also correlated to the conventional clinicopathological factors in this research.

## Methods

### Patients and treatment

**Fig. 1:**
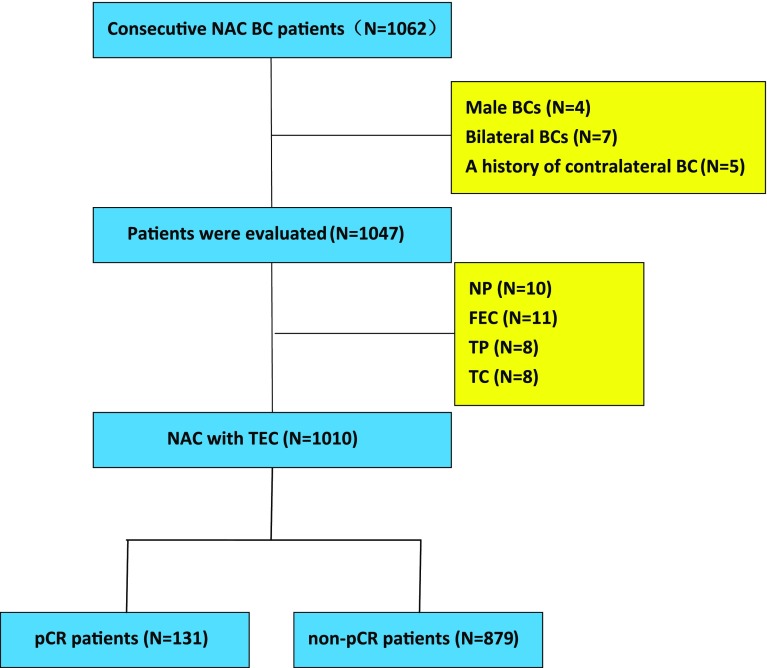
Chemo-effects and patient registration. Included and excluded in the analysis according to the availability of tumor material and treatment group (*E* epirubicin, *T* docetaxel, *C* cyclophosphamide, *F* fluorouracil, *N* vinorelbine, *P* Cisplatin)

Overall, 1062 consecutive patients with primary BC who were treated with both NAC and surgery at Breast Cancer Center of Chongqing at The First Affiliated Hospital of Chongqing Medical University from January 2012 to July 2017 were recruited in this retrospective research. Only patients who received ≥ 3 courses of treatment with TEC were included, which included intravenous administration of cyclophosphamide (500 mg/m^2^), epirubicin (75 mg/m^2^), and docetaxel (75 mg/m^2^) each 21 days. Other exclusion criteria included bilateral BC, male BC, and a history of contralateral BC, and finally 1010 patients were enrolled (Fig. 1). All HER-2 (+) patients were thoroughly informed of the effect of targeted therapy on the outcomes. However, only 3% (11/356) HER-2 (+) patients accepted trastuzumab due to financial reasons. All 11 patients received 4 cycles of EC regimen, then followed by T+ trastuzumab every 3 weeks (loading dose of 8 mg per kg of bodyweight infused intravenously during 90 min, followed by 6 mg/kg during 30 min every 3 weeks). After chemotherapy, they also received additional cycles of trastuzumab until 1 year. Furthermore, patients treated by trastuzumab monitored left ventricular ejection fraction (LVEF) at the beginning and end of chemotherapy, and none of them developed a heart complication (the absolute drops of LVEF were 0–7%).

This research was authorized by the ethics committee of The First Affiliated Hospital of Chongqing Medical University. All patients received and agreed with the informed consent.

### Immunohistochemical staining and intrinsic subtypes

ER, PR, HER-2 status, and Ki-67 index were measured before and after NAC by IHC. All results were evaluated independently by two pathologists. If more than 1% of nuclei were colored, we considered that ER and PR were positive [13]. If the specimen either recorded 3+ by IHC, or demonstrated an over 2.2-fold growth in fluorescence in situ hybridization (FISH), it could be regarded as HER2-positive (Fig. 1S). Tumor subtypes were defined based on the expression of ER, PR, and HER2: luminal (ER+ and/or PR+, HER2−), luminal–HER2 (ER+ and/or PR+, HER2+), HER2-rich (ER− and PR−, HER2+), and triple negative (ER− and PR− and HER2−). The Ki-67 value was explained as the proportion of positive cells (500–1000) with nuclear staining in the invasive front of the tumor as advised by the International Ki67 in Breast Cancer Working Group [14].

### Evaluation of the response to chemotherapy

Clinical diagnostic imaging (ultrasonography and magnetic resonance imaging) was utilized to measure the reaction of BC during NAC. Clinical response was specified by making the comparison of the alteration of primary lesions. Physical and imaging examinations based on Response Evaluation Criteria in Solid Tumors (RECIST) guidelines version 1.1 were utilized to assess treatment response as follows: cPR reduction in a total of target lesion diameters ≥ 30%; cPD growth in a total of target lesion diameters ≥ 20%; cSD neither sufficient reduction as cPR nor sufficient growth as cPD. Pathologic complete response (pCR) was explained as no remaining invasive disease in any excised breast tissue irrespective of nodal involvement [9].

### Statistical analysis

The comparison among quantitative characteristics was made by Kruskal–Wallis test, and the comparison among categorical features was displayed by *χ*^2^ test. Receiver operating characteristic (ROC) curve analysis was employed to measure the cut-off value of Ki67 indication. The whole of the analyses was conducted through SPSS version 22.0 (SPSS Inc, Chicago, USA). In this study, *P* value less than 0.05 was regarded as significant in statistical respect and every *P* value was two-sided.

## Results

### Baseline characteristics

One thousand and ten females with primary BC were inclusive. Chemotherapy was organized for a median of 4 cycles (range 3–8 cycles) before operation. The median age of the enrolled patients was 49 ± 8.485 years (range 20–72 years), and 39.5% of them were menopausal. The mean tumor size before and after NAC were 4.0 ± 3.89 and 2.12 ± 1.98 cm, respectively, and 41.0% of them had node-positive disease at diagnosis. One hundred and thirty-one patients (13.0%) achieved pCR, and 574 patients (56.8%) showed clinical response to NAC based on the RECIST criteria, while 305 patients (30.4%) showed no response, including 290 of cSD (28.7%) and 15 of cPD (1.5%). Patients, tumor, and treatment baseline characteristics as well as tumor reaction are detailed in Table 1.

**Table 1:** Clinicopathological features of the study cohort (n = 1010)

Parameters	Number (%)
Age (year)	
< 40	133 (13.2)
≥ 40	877 (86.8)
Menopause	
Yes	399 (39.5)
No	611 (60.5)
Chemotherapy cycles	
3	29 (2.9)
4	900 (89.2)
5–8	81 (7.9)
Subtypes of cancer	
Ductal	986 (97.6)
Lobular	13 (1.3)
Others	11 (1.1)
Tumor size	
< 2 cm	74 (7.3)
2–4 cm	522 (51.7)
≥ 4 cm	414 (41.0)
Clinical nodal status	
Positive	473 (46.8)
Negative	537 (53.2)
Histological grade	
I	26 (2.6)
II	578 (57.3)
III	130 (12.9)
Unknown	276 (27.2)
Response evaluation	
pCR	131 (13.0)
cPR	574 (56.8)
cSD	290 (28.7)
cPD	15 (1.5)
Responder (pCR and cPR)	705 (69.8)
Non-responder (cSD and cPD)	305 (30.2)

*pCR* pathological complete response, *cPR* clinical partial response, *cPD* clinical progressive disease, *cSD* clinical stable

66.3% of the whole cases showed ER-positive (31 cases with 1–10% positive) and 52.8% of the examined samples demonstrated PR positivity (20 cases with 1–10% positive). 35.2% of cases provided the evidence of HER2 positivity, and 70.8% of all patients had the expression of Ki67 ≥ 14%. Within this study, 36.8% (*n* = 372) were categorized as luminal type, 16.8% (*n* = 170) as luminal–HER2, 18.4% (*n* = 186) as HER2-rich, and 14.1% (*n* = 142) as triple negative. The Immunochemical data and molecular subtype features are shown in Table 2.

**Table 2:** Immunochemical data and molecular subtype features of the cohort (*n* = 1010)

Parameters	Number (%)
ER positivity score^a^	
0	340 (33.7)
0–10%	31 (3.1)
≥10%	639 (63.2)
PR positivity score^a^	
0	477 (47.2)
0–10%	20 (2.0)
≥ 10%	513 (50.8)
Her-2	
Positive	356 (35.2)
Negative	514 (50.9)
Unknown	140 (13.9)
Ki67	
< 14%	295 (29.2)
≥ 14%	715 (70.8)
Molecular subtype^b^	
Luminal	372 (36.8)
Luminal/HER2	170 (16.8)
HER2	186 (18.4)
TN	142 (14.1)
Unknown	140 (13.9)

*ER* estrogen receptor, *PR* progesterone receptor, *HER2* human epidermal growth factor receptor 2

^a^Positive ≥ 1%

^b^Luminal: HR+/HER2−, luminal/HER2: HR+/HER2+, HER2: HR−/HER2+, TN: HR−/HER2−

### Association between baseline characteristics and clinical or pathological response to NAC

Chi-square test (*χ*^2^) was utilized to assess the relationship between the clinical pathological parameters and the clinical or pathological response to NAC. As shown in Table 3, Age and HER2 status did not demonstrate a significant response to NAC in statistical results (all *P* *>* 0.05). Patients with greater Ki67 level (≥ 14%) had better clinical and pathological response to NAC (*P* < 0.001). In the clinical response assessment, patients with positive ER and PR status did not show significance in statistical respect (*P* = 0.904 and *P* = 0.542, separately) while the tumor size (≥ 4 cm) and positive clinical nodal were significantly associated with tumor size reduction (*P* < 0.001 and *P* = 0.03, respectively), and the menstrual status had marginal P values (P = 0.058). In the pathological response assessment, patients with negative ER and PR status were more likely to achieve pCR (both *P* < 0.001). Furthermore, the tumor diameter (< 4 cm) was significantly connected with pCR (P = 0.038), whereas the menstrual and clinical nodal status did not show statistical significance (*P* = 0.962 and *P* = 0.289, separately).

**Table 3:** Clinicopathological characteristics of pre-NAC according to clinical and pathological response

Characteristic	Clinical response			Pathology response		
	cRes	Non-cRes	*P* value	pCR	Non-pCR	*P* value
Age (year)			0.384			0.466
< 40	95	35		20	113	
≥ 40	610	270		111	766	
Menopause			0.058			0.962
Yes	265	134		52	347	
No	440	171		79	532	
Tumor size (cm)			< 0.001			0.039
< 4	391	205		88	505	
≥ 4	314	100		43	371	
Clinical nodal status			0.03			0.289
Yes	346	127		67	406	
No	359	178		64	473	
ER status			0.904			< 0.001
Positive	442	190		56	576	
Negative	263	115		75	303	
PR status			0.542			< 0.001
Positive	332	150		32	450	
Negative	373	155		99	429	
Her2 status			0.092		318	0.233
Positive	270	96		48		
Negative	348	166		54	460	
Ki67 expression (%)			< 0.001			< 0.001
< 14	182	113		16	279	
≥ 14	523	192		115	600	

*cRes* pCR and cPR, *Non-cRes* cPD and cSD

In addition, the response to NAC between patients with weakly hormone receptor (HR) (1–10% positive) and HR ≥ 10% or HR− were also assessed. As shown in Fig. S2, patients with HR 1–10% positive did not demonstrate a significant response to NAC in statistical results compared with HR ≥ 10% or HR− patients (all *P* *>* 0.05).

### Evaluation of the predictive value of pretreatment and decreased Ki67 during NAC in different molecular subtypes

**Fig. 2:**
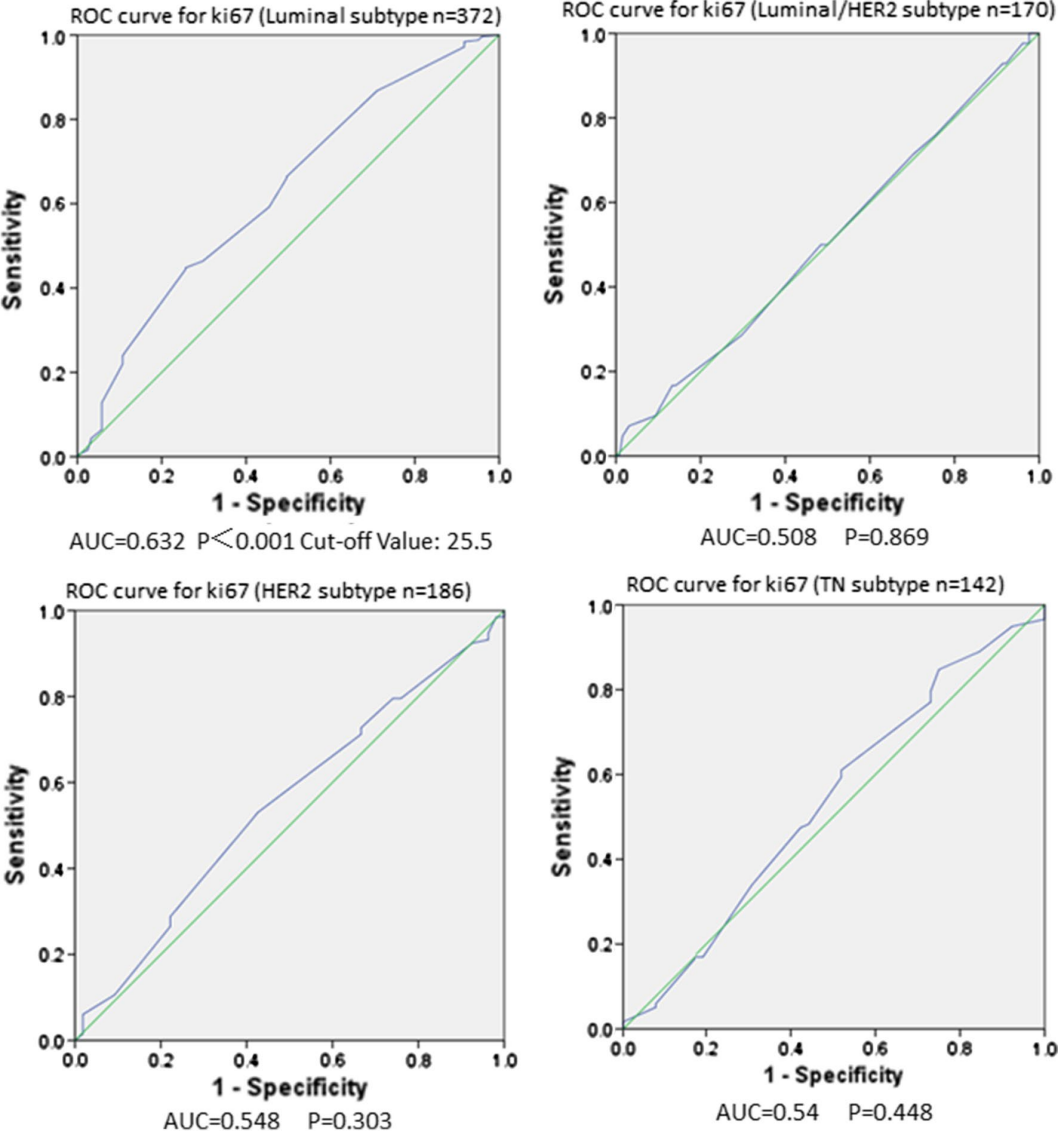
Comparison of the predictive role of pre-NAC Ki67 for the response to NAC by ROC curve analysis among the different molecular subtypes

**Fig. 3:**
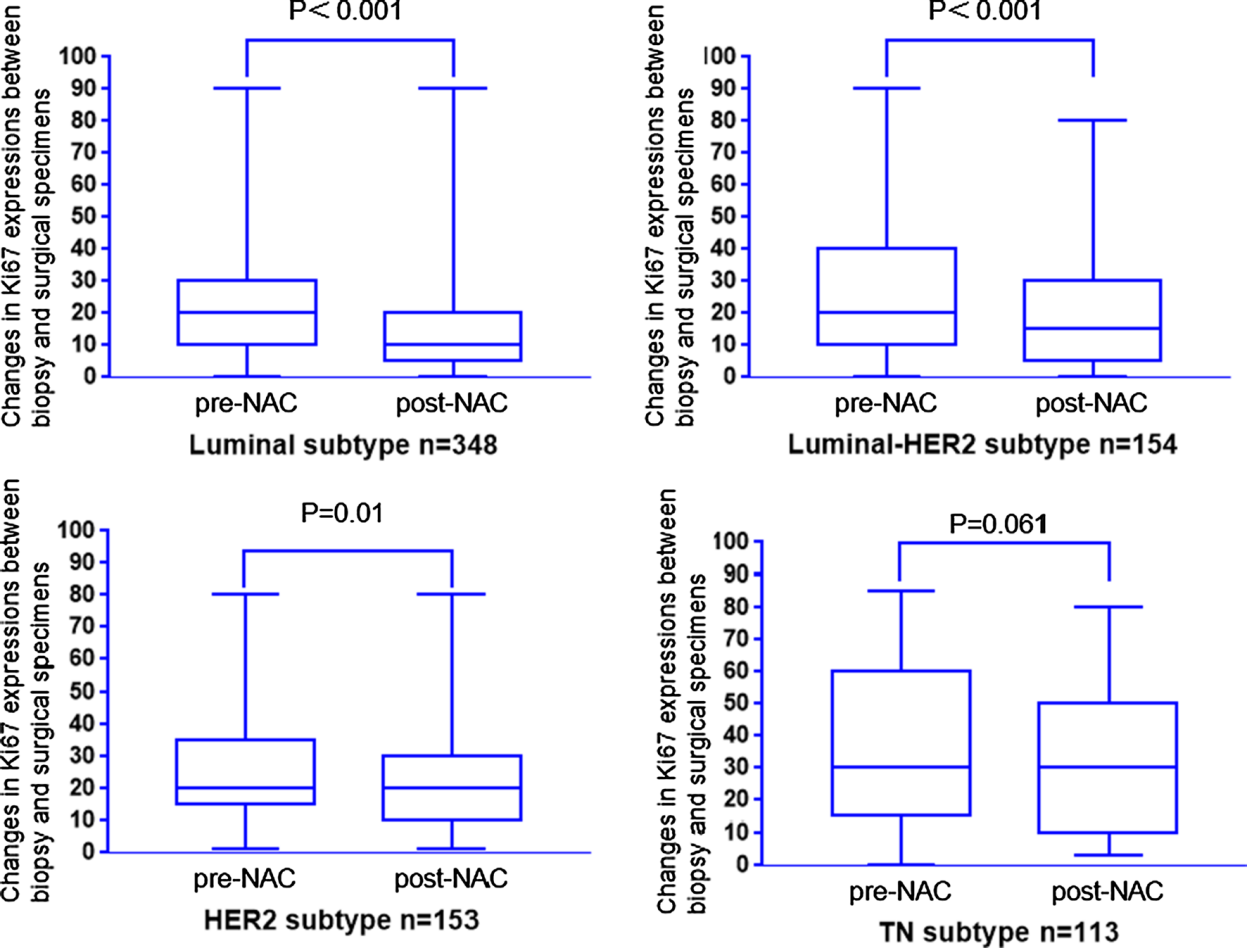
Changes in Ki67 expression between biopsy and surgical specimens in the different molecular subtypes

ROC curve analysis was utilized to identify the predictive value of pretreatment Ki67 expression response to NAC in different molecular subtypes (Fig. 2). Area under ROC curve (AUC) of Ki67 expression was 0.632 in luminal-type BCs (*P* < 0.001, 95% CI 0.565–0.686). On the contrary, the AUC of Ki67 expression were 0.508, 0.548, and 0.54 in luminal–HER2, HER2-rich, and triple-negative type BCs separately, demonstrating that Ki67 level according to biopsy specimen was ineffective in forecasting of therapeutic response among these subtypes (*P* = 0.869, *P* = 0.303, and *P* = 0.448, respectively). Accordingly, the result of ROC curve analysis showed that pretreatment Ki67 expression could not be used as a predictor of NAC in luminal/HER2 subtypes with HR 1–10% positive (*P* = 0.357, Fig. 3S). Furthermore, in luminal-type BCs, we recognized that 25.5% as the best cut-off value of pretreatment Ki67 for predicting response to NAC with an ideal sensitivity of 46.6% and a specificity of 69.9%. On the other hand, the positive predictive value and negative predictive value were 78.5 and 39.2%, respectively.

**Fig. 4:**
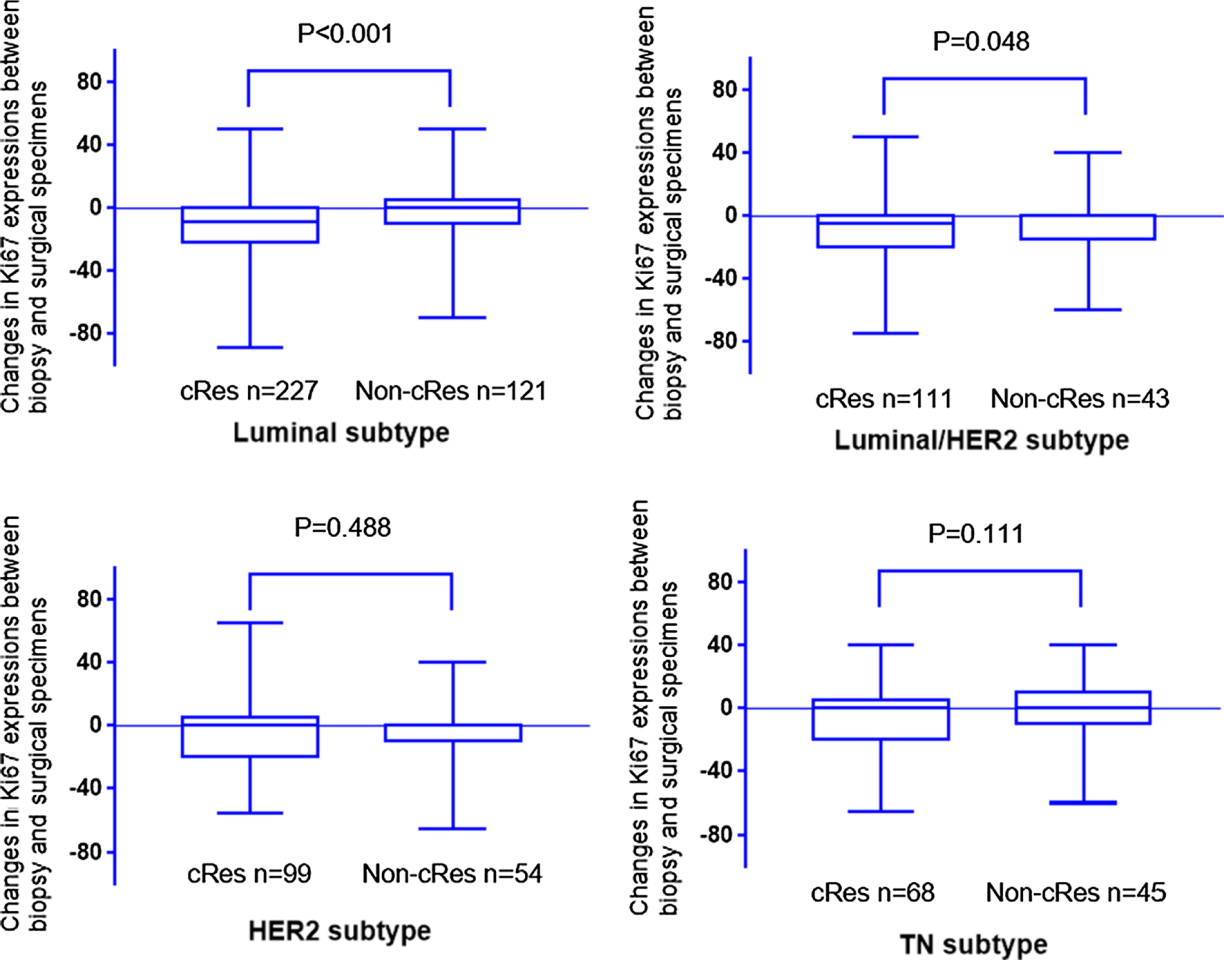
Changes in Ki67 expression between biopsy and surgical specimens according to patient outcomes among the different molecular subtypes

To assess the changes in Ki67 during NAC, differences in Ki67 expression between biopsy and surgical specimens from the same patient were evaluated in 879 patients. As shown in Fig. 3, post-NAC Ki67 levels were greatly reduced in luminal, luminal–HER2, and HER2-rich subtypes (*P* < 0.001, *P* < 0.001, and *P* = 0.01, separately), and the triple-negative type BCs had marginal *P* values (*P* = 0.061). We also explored the association between tumor size reduction and the decrease of Ki67 in different molecular subtypes (Fig. 4). In our analysis, tumor size reduction was closely related to the decrease of Ki67 during NAC in luminal and luminal–HER2 subtypes (*P* < 0.001 and *P* = 0.048, separately). On the contrary, Insignificant statistical distinctions in the decrease of Ki67 during NAC were identified between the cRes and non-cRes group in HER2-rich and triple-negative subtypes (*P* = 0.488 and *P* = 0.111, separately).

## Discussion

NAC, which downstages the disease and reduces tumor volume, has been resulted in improved success rates of breast-conserving operations. Furthermore, the patients who attain pCR after NAC have a very low risk of relapse and death regardless of the earlier stage and molecular subtype [4, 15]. In the latest years, it has been accepted that BC can be categorized into multiple subtypes by IHC analysis of ER, PR, HER-2, and Ki67, and several studies revealed the response to NAC in different molecular subtypes [1, 3, 5, 6]. However, some of these conclusions remain controversial [11, 16].

A great number of studies have demonstrated a positive connection between Ki67 expression and chemotherapy response [2, 8, 17, 18]. In some studies, Ki67 is thought to predict NAC response only in ER-positive BCs [2, 18–20]. However, some clinical trial with relatively large cases suggested that Ki67 independently improved the prediction of treatment response in luminal tumors as well as triple-negative tumors [8, 17, 21]. Furthermore, although some studies revealed that Ki67 changes play different predictive roles in ER-positive and ER-negative patients during NAC, the limited number of cases limits further typing analysis [12, 22].

In this study, we first assessed the usefulness of commonly applied tumor characteristics to forecast clinical response and pCR after NAC. In the clinical response assessment, pretreatment Ki67 (≥ 14%), tumor size ≥ 4 cm, and positive clinical nodal were significantly associated with tumor size reduction, whereas Age, ER, PR, and HER2 status did not show a statistically significant response to NAC. In addition, the menstrual status had marginal P values in this analysis. Furthermore, patients with negative ER, PR, and smaller tumor size (< 4 cm) were more probable to attain pCR, while the Age, HER2 status, menstrual, and clinical nodal status did not show statistically significant response in pathological response assessment. Moreover, the response to NAC of patients with HR 1–10% positive were also evaluated because recent research indicated that weakly HR expression was a poor correlate of luminal subtype [23], and the therapeutic effect of hormone therapy of patients with HR 1–10% positive was poor [24]. According to our results, patients with HR 1–10% positive did not demonstrate a significant response to NAC in statistics compared with HR ≥ 10% or HR– patients. However, the number of patients with HR 1–10% positive in our study was relatively small (*n* = 31), so it is necessary to further confirm the above relationship through large sample studies.

Most of the above results were in line with previous literature [12, 16, 19, 25]. However, the HER2 status did not show a statistically significant response in clinical and pathological response in our analysis, which is not consistent with the existing literature. The possible explanation is that only 3% HER2 (+) patients accept trastuzumab before surgery in this study, while this phenomenon also confirms the importance of using trastuzumab in HER2 (+) patients during NAC [26, 27].

The overall pCR rate in our study was 13%, which was relatively low compared with some large studies (15.8–27.1%) [9, 28, 29]. We speculated that this phenomenon may be due to relatively short cycles of NAC in this study (89.2% of patients experienced 4 cycles of chemotherapy) compared with the 8 cycles of NAC in other studies [9, 29]. Another possible reason for this is that the proportion of patients with clinical tumor size ≥ 4 cm in our study reached 41%, which was significantly higher than the 28% of NSABP B-27 trial [15] because NAC may aid in shrinking the size of the tumor instead of directly leading to pCR status [30].

Next, we focused on evaluating the predictive role of Ki67 expression in different molecular subtypes for response to NAC. According to our findings, the pretreatment Ki67 could be used as a predictor of NAC only in luminal-type BCs. With a ROC curve, we identified 25.5% as the best cut-off value of pretreatment Ki67 for predicting response to NAC with an optimal sensitivity of 46.6% and a specificity of 69.9%. To assess the impact of Ki67 changes on response to treatment, we first investigated the changes in Ki67 after NAC in different molecular subtypes. As expected, a decrease in Ki67 had been found in almost all molecular subtypes after NAC (the triple-negative type BCs had a borderline significant), which was consistent with previous research [18]. However, when considering the distribution of responders and non-responders, the reduction in Ki67 may induce a positive response only in luminal and luminal–HER2 subtypes.

Our results show that Ki67 has been demonstrated to forecast NAC response only in ER-positive BCs, which are consistent with several studies [2, 12, 17, 18, 20]. Furthermore, our research also revealed that the reduction in Ki67 could not be used as a predictor of NAC in HER2-rich and triple-negative subtypes, which was in line with previous study [12]. It has been shown that the post-treatment, but not pretreatment Ki67 indication levels identify a group of patients at great danger for relapse in patients who do not achieve pCR [9]. Therefore, patients with HER2-rich and triple-negative subtype who exhibit marked tumor shrinkage in NAC but still have high levels of Ki67 might be good candidates for more aggressive adjuvant systemic treatments, such as extension of postoperative chemotherapy, proper radiotherapy, and administration of Capecitabine, which was recently shown to prolong the survival of non-pCR patients [31]. Another interesting finding in this study is that the response to NAC in patients with luminal–HER2 subtype can be predicted by the reduction in Ki67, but not by pretreatment Ki67. Possible explanations for this phenomenon are tumor heterogeneity [32], different signaling pathways caused by chemotherapy [33], and the presence of more molecular subtypes.

The major limitation of this study is that our data come from a retrospective study in one single center. Therefore, it is necessary to perform prospective study to obtain evidence supporting our results.

## Conclusion

This analysis suggested that the pretreatment and decreased Ki67 play different predictive roles in different molecular subtypes treated with NAC. The best cut-off for pretreatment Ki67 in predicting the connection with the tumor size decrease was 25.5% in luminal subtype. In addition, aggressive adjuvant systemic treatments should be considered for patients with HER2-rich and triple-negative subtype who exhibit tumor shrinkage in NAC but still have high levels of Ki67.

## Electronic supplementary material

Below is the link to the electronic supplementary material.

Supplementary material 1 (DOCX 8175 kb)

Supplementary material 2 (DOCX 65 kb)

Supplementary material 3 (DOCX 31 kb)
