# Supplementary material for: Assessment of the predictive role of pretreatment Ki-67 and Ki-67 changes in breast cancer patients receiving neoadjuvant chemotherapy according to the molecular classification: a retrospective study of 1010 patients
Source: Breast Cancer Res Treat. 2018 Feb 26;170(1):35–43. doi: 10.1007/s10549-018-4730-1 (PMC5993857; doi:10.1007/s10549-018-4730-1)

**Fig. 1S** **Representative figure of FISH for patients with Her2 IHC staining 2+ (The HER2/CEP17**

**ratio in the left and right were 1.21 and 3.18 respectively).**


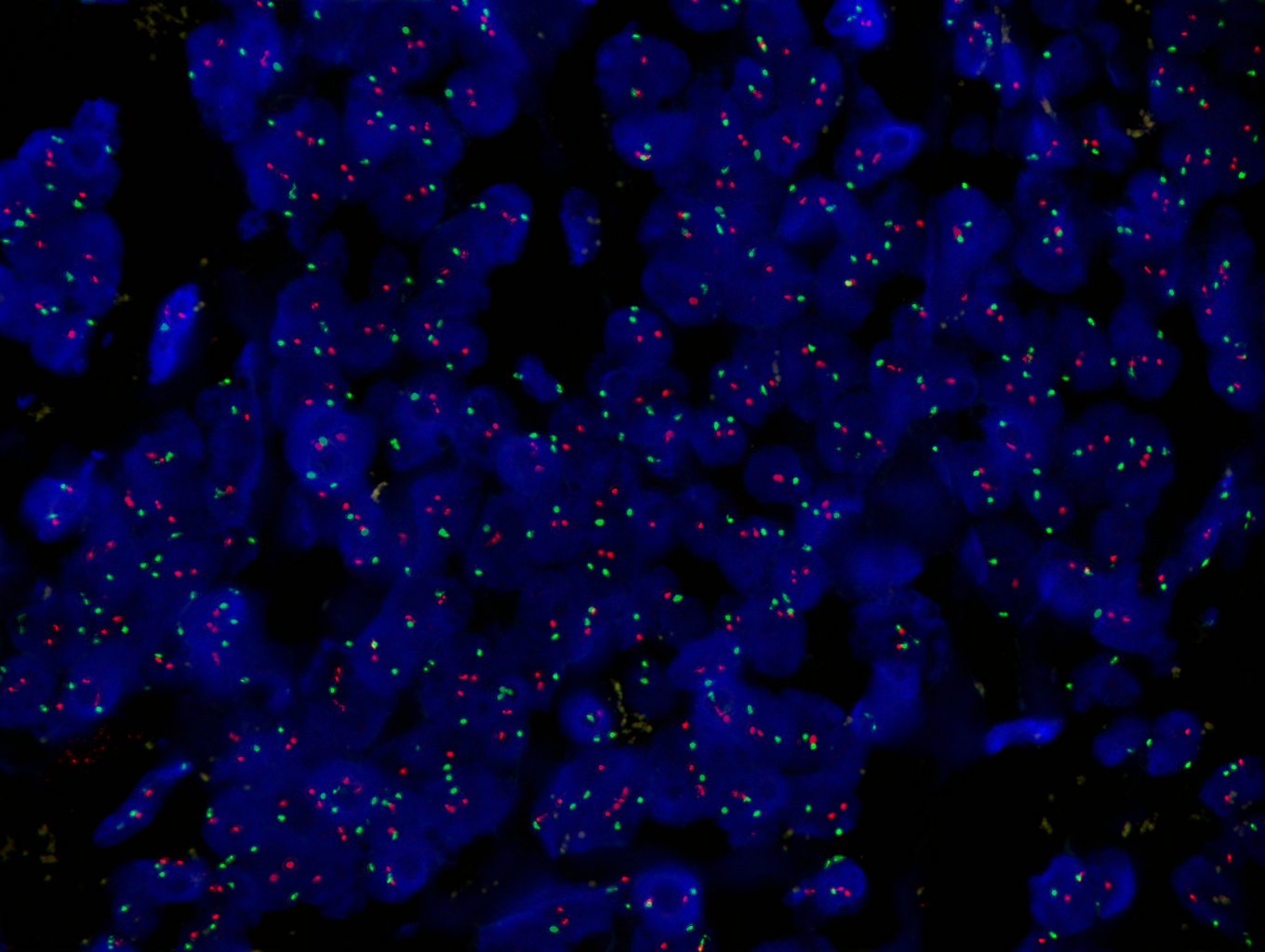

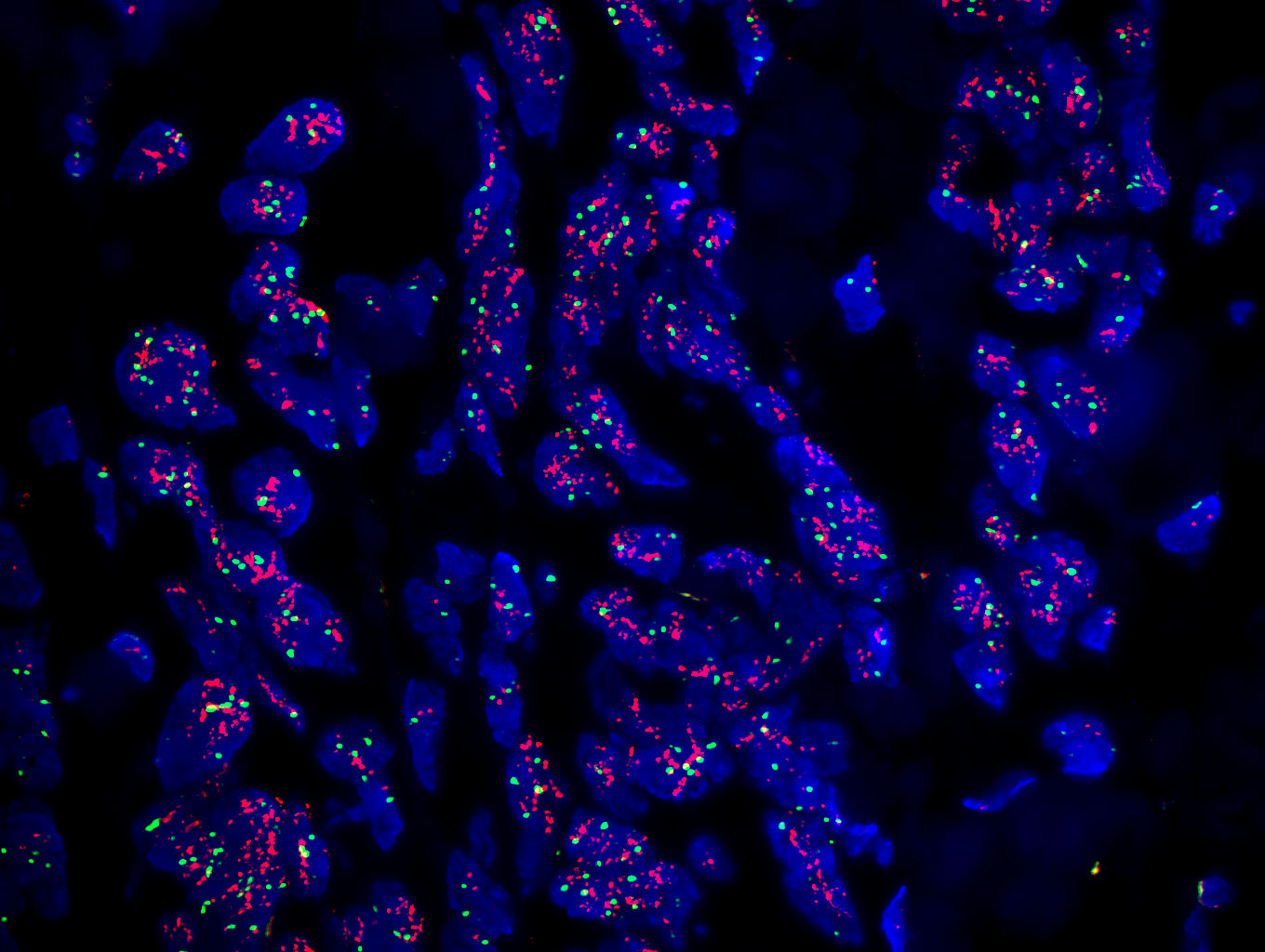

Supplement: Supplementary file 1 — Supplementary material 1 (DOCX 8175 kb) [file 10549_2018_4730_MOESM1_ESM.docx]
