# Supplementary material for: Assessment of the predictive role of pretreatment Ki-67 and Ki-67 changes in breast cancer patients receiving neoadjuvant chemotherapy according to the molecular classification: a retrospective study of 1010 patients
Source: Breast Cancer Res Treat. 2018 Feb 26;170(1):35–43. doi: 10.1007/s10549-018-4730-1 (PMC5993857; doi:10.1007/s10549-018-4730-1)

**Fig. 2S** **Comparison of the remission rates between HR+（1-10%positive）subtypes and HR+（****≥10%positive）subtypes or HR- patients to NAC.**


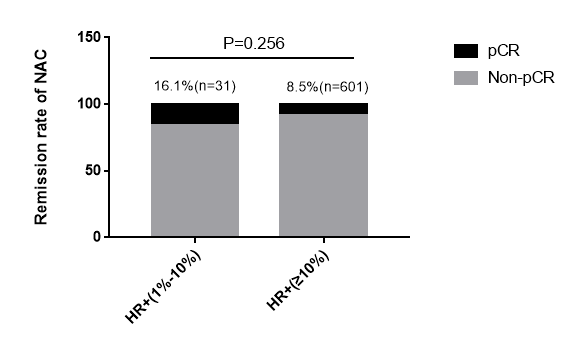

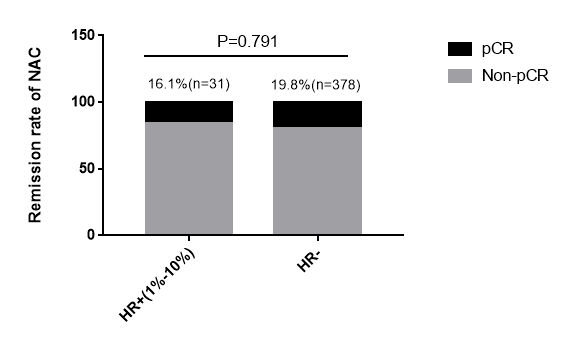

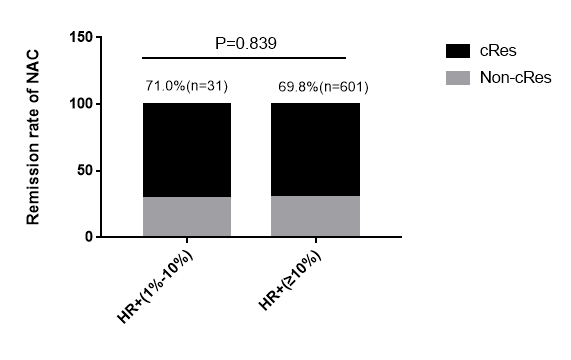

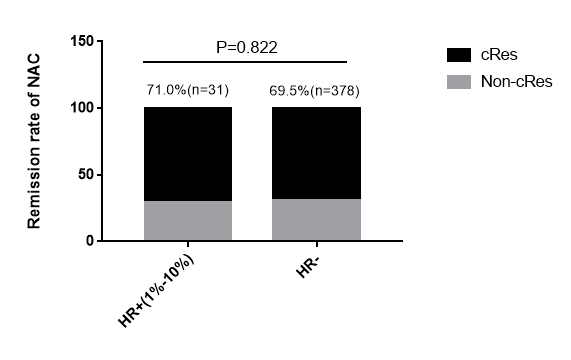

Supplement: Supplementary file 2 — Supplementary material 2 (DOCX 65 kb) [file 10549_2018_4730_MOESM2_ESM.docx]
