# Supplementary figures and images for: Assessment of the predictive role of pretreatment Ki-67 and Ki-67 changes in breast cancer patients receiving neoadjuvant chemotherapy according to the molecular classification: a retrospective study of 1010 patients
Source: Breast Cancer Res Treat. 2018 Feb 26;170(1):35–43. doi: 10.1007/s10549-018-4730-1 (PMC5993857; doi:10.1007/s10549-018-4730-1)

**Fig. 3S** **The predictive role of pretreatment ki67 by ROC curve in** **luminal/HER2 (HR1-10% positive） subtypes**


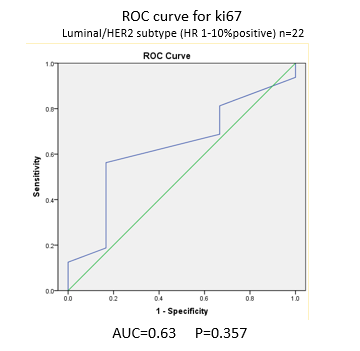

Supplement: Supplementary file 3 — Supplementary material 3 (DOCX 31 kb) [file 10549_2018_4730_MOESM3_ESM.docx]
